# DIVAS: an R package for identifying shared and individual variations of multiomics data

**DOI:** 10.1093/bioinformatics/btag615

**Published:** 2026-08-18

**Authors:** Yinuo Sun, James Stephen Marron, Kim-Anh Lê Cao, Jiadong Mao

**Affiliations:** Melbourne Integrative Genomics, School of Mathematics and Statistics, The University of Melbourne, Parkville, Victoria 3010, Australia; Department of Biochemistry and Molecular Biology, Cancer and Infection Programs, Biomedicine Discovery Institute, Monash University, Clayton, Victoria 3800, Australia; Department of Statistics and Operations Research, The University of North Carolina at Chapel Hill, Chapel Hill, NC 27599, United States; School of Data Science and Society, The University of North Carolina at Chapel Hill, Chapel Hill, NC 27599, United States; Melbourne Integrative Genomics, School of Mathematics and Statistics, The University of Melbourne, Parkville, Victoria 3010, Australia; Melbourne Integrative Genomics, School of Mathematics and Statistics, The University of Melbourne, Parkville, Victoria 3010, Australia

## Abstract

**Motivation:**

Multiomics data integration aims to identify biological patterns shared across molecular modalities. Most existing methods detect either jointly shared variation, across all modalities, or individual variation, unique to a single modality, but overlook partially shared variation, shared by only a subset of modalities. This is a critical limitation, because many biological mechanisms manifest in some but not all molecular modalities.

**Results:**

We present an open-source R package implementing data integration via analysis of subspaces (DIVAS), a framework for systematically identifying jointly shared, partially shared and individual variations across multiple data types. DIVAS combines angle-based subspace analysis with inference through rotational bootstrap, hierarchically searching all combinations of modalities to decompose multiomics data into interpretable components with scores and loadings. In simulations with a known sharing structure, DIVAS recovered every component across a wide range of noise levels, whereas existing methods did not. Applied to multi-modal COVID-19 data, it reveals partially shared immune and metabolic dysregulation patterns underpinning disease severity that conventional approaches would miss.

**Availability and implementation:**

DIVAS is available at https://github.com/ByronSyun/DIVAS, with documentation and vignettes. The COVID-19 case study vignette is available at https://byronsyun.github.io/DIVAS_COVID19_CaseStudy/.

## 1 Introduction

Modern high-throughput technologies measure diverse molecular layers, from genomics to metabolomics, on the same biological samples. These multiomics datasets offer complementary views of biological processes, yet analyzing them in a unified framework remains challenging. The key question is how to systematically identify which biological signals are shared across all omics types (*jointly shared*), which are shared by only some (*partially shared*) and which are unique to a single layer (*individual*).

Most existing multivariate approaches identify only jointly shared variation. Methods based on canonical correlation analysis (CCA) or partial least squares find lower-dimensional representations by maximizing correlations between input data blocks; widely used examples include regularized CCA (RCCA) (Tenenhaus and Tenenhaus 2011), sparse RCCA (Tenenhaus *et al.* 2014), regularized generalized CCA (RGCCA) (Tenenhaus *et al.* 2017), and DIABLO (Singh *et al.* 2019).

Joint and individual variation explained (JIVE) (Lock *et al.* 2013) decomposes multiomics data into joint variation shared across all data blocks and individual variation unique to each. Angle-based JIVE (AJIVE) (Feng *et al.* 2018) improved on JIVE using principal angle analysis, giving faster joint structure selection and stronger theoretical justification through matrix perturbation theory.

Multi-omics factor analysis (MOFA) (Argelaguet *et al.* 2018) and MOFA+ (Argelaguet *et al.* 2020) use Bayesian group factor analysis to identify latent factors explaining variation within and across omics layers, and began to explore partially shared variation. However, they do not systematically enumerate all possible patterns of partial sharing, and the interpretation of which subset of blocks shares each factor can be ambiguous.

A parallel line of work uses deep generative models, such as MultiVI (Ashuach *et al.* 2023) and MOVE (Allesøe *et al.* 2023). These target nonlinear representation learning, imputation, batch correction, and prediction, returning a learned latent embedding rather than a decomposition labeled by which modalities share each signal; they are, therefore, complementary to the methods considered here.

Recent methods target partially shared variation directly. Structural learning and integrative decomposition (SLIDE) (Gaynanova and Li 2019) models variation shared between specific subsets of blocks using structured sparsity, with block-specific patterns determined by bi-cross-validation, but it does not systematically detect all patterns of partial sharing and lacks statistical inference for those it finds. Hierarchical nuclear norm (HNN) penalization (Yi *et al.* 2023) formulates partially shared variation through hierarchical levels, using a convex formulation that avoids scores-loadings factorization; it offers identifiability guarantees but requires computationally intensive tuning parameter selection. No package is available for either method.

Table 1 summarizes these methods. The critical gap is the systematic identification of partially shared variation, which matters most with three or more modalities: there is no guarantee that the most interesting biological patterns are captured by jointly shared variation alone. A regulatory mechanism may affect transcriptomics and proteomics but not metabolomics, while metabolic dysregulation may appear in metabolomics and proteomics without being reflected transcriptionally.

**Table 1:** Comparison of multiomics integration methods.

Method	Jointly shared	Partially shared	Individual	Statistical inference	Package availability
RCCA/RGCCA	✓	✗	✗	✗	CRAN^h^
DIABLO	✓	✗	Limited^a^	✗	Bioconductor^i^
JIVE	✓	✗	✓	Limited^b^	CRAN^j^
AJIVE	✓	✗	✓	Limited^c^	GitHub (Carmichael 2020)
MOFA/MOFA+	✓	Limited^d^	✓	✓^e^	Bioconductor^k^
SLIDE	✓	✓	✓	Limited^f^	N/A
HNN	✓	✓	✓	Limited^g^	N/A
DIVAS	✓	✓	✓	✓	GitHub

“✓” indicates that a method is capable of performing a specific task, while “✗” denotes that the method is not equipped to handle the task. The capabilities of DIVAS, MOFA+, and AJIVE are additionally assessed empirically in Supplementary Methods Section 3, available as supplementary data at *Bioinformatics* online.

a Focus on joint variation; individual variation not explicitly modeled.

b Permutation testing for rank selection only.

c Angle bounds for joint versus individual separation, but not for rank selection.

d Learns sparsity patterns but does not detect all partial sharing patterns; the interpretation of shared patterns can be ambiguous.

e Bayesian inference on factors and loadings.

f Cross-validation for model selection only.

g Convex optimization with tuning parameter selection via bi-cross-validation.

h https://CRAN.R-project.org/package=RGCCA.

i https://www.bioconductor.org/packages/release/bioc/html/mixOmics.html.

j https://CRAN.R-project.org/package=r.jive.

k https://www.bioconductor.org/packages/release/bioc/html/MOFA2.html.

Addressing this gap, data integration via analysis of subspaces (DIVAS) was proposed by Prothero *et al.* (2024) (see also Ackerman *et al.* (2025) for an application in neuroimaging). The framework systematically identifies jointly shared, partially shared and individual variations, provides inference procedures with statistical guarantees, and uses broadly useful defaults enabling fully data-driven integration without manual tuning.

Until now, however, DIVAS has been available only as closed-source MATLAB code, limiting its uptake. The statistical methodology is that of Prothero *et al.* and is not modified here; the contribution of the present work is software: (i) an open-source R implementation of the full algorithm; (ii) visualization and downstream-interpretation tools not previously available; (iii) documentation and vignettes for users without a background in matrix perturbation theory or convex optimization; and (iv) the first quantitative benchmark of DIVAS against alternative methods on simulated data with a known sharing structure, together with an applied multiomic case study.

## 2 Methods

The input of DIVAS is a collection of *K* data blocks $X_{1},\ldots ,X_{K}$ from *N* samples, where each $X_{k}$ is a $\left(d_{k}\times N\right)$ feature-by-sample matrix. These data blocks represent multiomic measurements from the same samples. The DIVAS algorithm, detailed in Prothero *et al.* (2024), comprises the following two main steps:

1. denoising individual data blocks and
2. identifying shared and individual structures among denoised data blocks.

**Figure 1:**
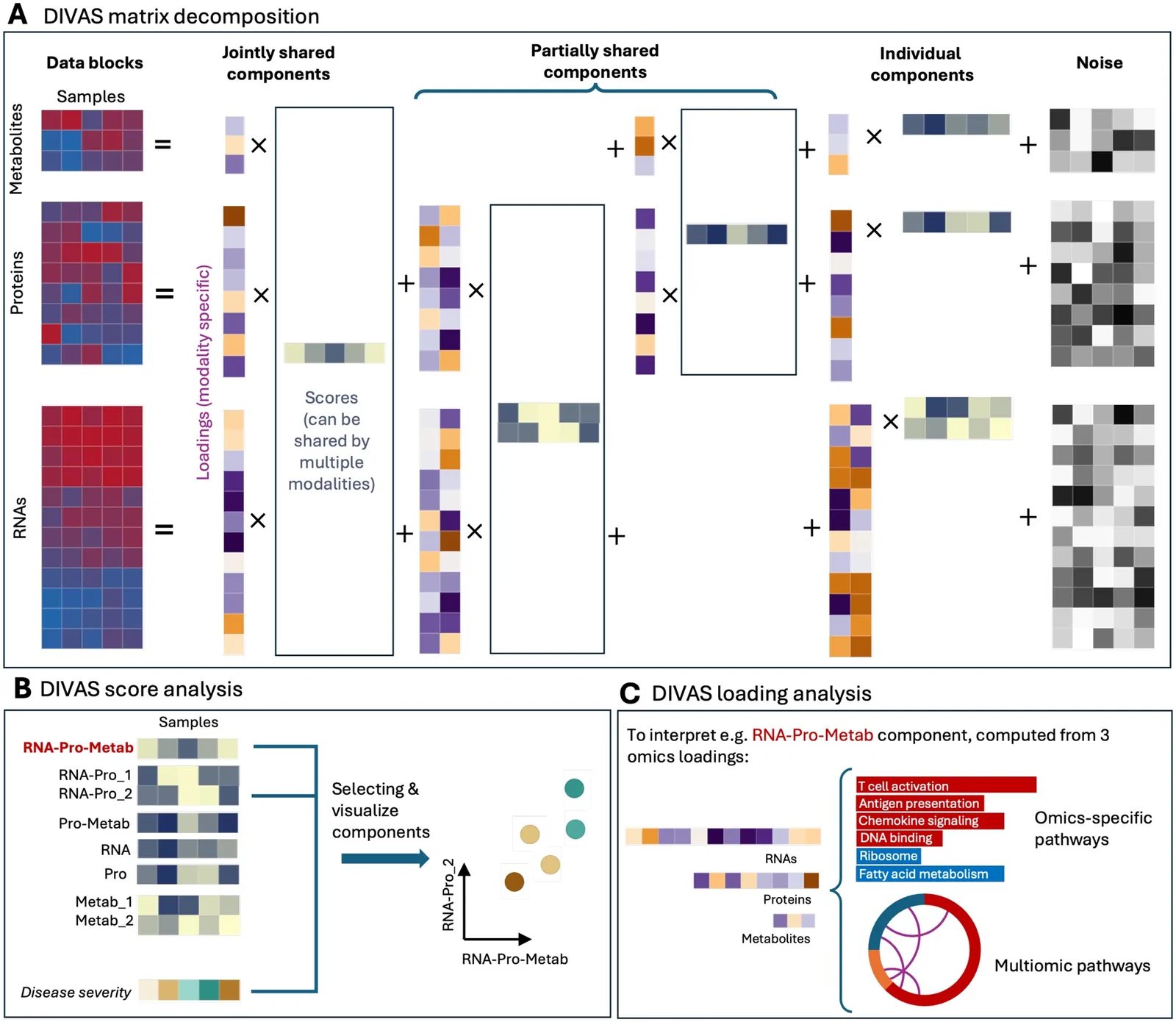
Schema of DIVAS. (a) DIVAS decomposes input data blocks into components representing jointly shared, partially shared, and individual modes of variation. Here we present a toy example taking transcriptomics, proteomics, and metabolomics as input. Each DIVAS component, representing a specific mode of variation, is defined by a score vector and one or more loading vectors (corresponding to one or more modalities). In the toy example, we found one three-way jointly shared component (RNA–protein–metabolite), three two-way partially shared components (two RNA–protein, one protein–metabolite), and four individual components (two RNA, one protein, one metabolite). (b) DIVAS scores are lower dimensional summaries of samples and can be used for visualization. For example, we can select the scores most highly correlated with disease severity and visualize them using a scatter plot. (c) DIVAS loadings measure the contribution of individual omics features to scores. Whereas one DIVAS component only has one common score vector, it may have multiple loading vectors, corresponding a set of data blocks. By identifying pathways represented by important omics features, we can interpret the biological meanings of individual components. In the notation of Section 2, each data block $X_{k}$ in (a) is $d_{k}\times N$, each loading matrix $L_{i,k}$ is $d_{k}\times r_{i}$, each score matrix $S_{i}^{⊤}$ is $r_{i}\times N$, and each noise matrix $E_{k}$ is $d_{k}\times N$. The rank decomposition and angle diagnostics are returned directly by DIVASmain; the score visualizations in (b) are produced by the companion vizOmics package, which takes DIVAS output objects as input; and for (c), DIVAS: getTopFeatures returns ranked feature tables that users pass to their preferred enrichment and plotting tools.

Both steps are fully data-driven and tuning free. The output is a set of components explaining shared and individual variations, each with scores and loadings as in standard factor models; see Fig. 1 for a schema.

### 2.1 Denoising data blocks

We assume that each data block has an underlying low rank structure $A_{k}$, which is contaminated by observational noise $E_{k}$ (Fig. 1a). That is, we assume $X_{k}=A_{k}+E_{k}$. To recover $A_{k}$ from noisy observation $X_{k}$, we apply principal component analysis to $X_{k}$ and take the first PCs. The number of PCs to use is determined by an optimal shrinkage method (Shabalin and Nobel 2013, Gavish and Donoho 2017) in a fully data-driven way.

### 2.2 Identifying shared and individual structures

We assume that the total variation in each $A_{k}$, the noiseless version of the *k*th data block, can be decomposed into jointly shared, partially shared and individual variations. That is, with i denoting a subset of the index set of all data blocks {1,…,K}, we assume that


$$A_{k}=\sum_{\text{all}\;\text{the}\;i’s\;\text{that}\;\;\text{contain}\;k}L_{i,k}S_{i}^{⊤},$$

where $S_{i}$ is an $\left(N\times r_{i}\right)$ score matrix, with $r_{i}$ denoting the number of components shared by data blocks in i, and where $L_{i,k}$ is a $\left(d_{k}\times r_{i}\right)$ loading matrix, linking the $r_{i}$ components with the $d_{k}$ features of the *k*th data block. That is, a DIVAS component shared by data blocks in i comprises *common* scores for all those data blocks, together with one loading vector for each data block in i; see Fig. 1a for an example involving three data blocks.

The core of the algorithm is the convex-concave optimization procedure of Prothero *et al.* (2024), of which we give only a sketch here. A shared structure represents a pattern of biological variation capturing how omics variables co-vary across samples, and finding it amounts to identifying the directions pointing toward the most important such patterns. DIVAS searches hierarchically, starting from the highest-order structures, shared among the most data blocks, and moving progressively to lower-order and individual structures. For each candidate direction it tests whether that direction stays close to the signal patterns of all included blocks while remaining sufficiently distant from the excluded ones, continuing until no further directions can be found for the current structure. The strength of this approach is that it determines automatically how many dimensions are needed for each type of relationship, giving a data-driven decomposition into interpretable shared and individual components.

### 2.3 Interpreting scores and loadings

The decomposition yields scores and loadings for each identified component. Each component has a score vector summarizing, in one dimension, how samples vary along that mode of variation, shared across all data blocks involved in the structure (Fig. 1a and b). A jointly shared component across transcriptomics, proteomics, and metabolomics, e.g. has a single common score vector.

Loadings link those shared scores to the features measured in each block. A component has one score vector but may have several modality-specific loading vectors (Fig. 1a and c), each indicating which features of that block contribute to the shared variation. For a jointly shared component, the transcriptomics loadings reveal which genes drive the pattern, while the proteomics and metabolomics loadings identify the associated proteins and metabolites. The top contributing features can then be taken forward for pathway analysis (Fig. 1c).

## 3 Results

### 3.1 Benchmarking on simulated data

We first assessed whether DIVAS recovers a known sharing structure, using three matched data blocks generated with one jointly shared, three partially shared and three individual rank-one components, over 10 noise levels and five random seeds (Supplementary Methods Section 3, available as supplementary data at *Bioinformatics* online). DIVAS identified the correct rank for all seven components, in every seed, up to a noise standard deviation of 2.00, and the chordal distance to the true score subspaces increased smoothly with noise (Figs 3 and 4, available as supplementary data at *Bioinformatics* online). On the same data, AJIVE over-estimated the rank of every component it returned and provides no partially shared components at all, while MOFA + factors, mapped *post hoc* to sharing subsets by their per-block variance explained, recovered the partially shared components in at most 4 of 50 datasets (Figs 5 and 6, available as supplementary data at *Bioinformatics* online).

### 3.2 Study design and data

We analyzed a multiomic dataset from a cohort of mild to severe COVID-19 patients (Su *et al.* 2020). The study included 139 patients measured at two time points, T1 and T2, giving 265 samples. Of the available omics types we selected single-cell RNA sequencing (scRNA-seq), bulk proteomics, and bulk metabolomics; 120 patients had all three, giving 240 samples across the two time points. Since DIVAS assumes independence between samples for inference, we avoided repeated measurements of the same subjects. We used stratified sampling to split the 120 patients into two groups of 60, retaining the T1 observation of one group and the T2 observation of the other, so that each patient contributes exactly one sample. After restricting to samples with complete measurements in all six data blocks, 114 samples remained.

DIVAS requires only that its input blocks are measured on the same samples in the same order; it does not require any particular type of block. Here, however, the scRNA-seq measurements are cell-level whereas the proteomics and metabolomics are sample-level. To harmonize this difference in granularity, we annotated cell types with CellTypist (Domínguez Conde *et al.* 2022, Xu *et al.* 2023) and pseudo-bulked CD4 T, CD8 T, CD14 monocyte, and natural killer (NK) cells within each sample, these being abundant peripheral blood mononuclear cell populations with robust annotations and important roles in the COVID-19 immune response (Su *et al.* 2020). The gene expression (GEX) profiles of these four cell types, together with proteomics (pro) and metabolomics (metab), formed six input blocks. Splitting a modality by cell type is a design choice specific to this case study, not a required preprocessing step: it lets DIVAS resolve programs shared across cellular compartments as well as across molecular modalities. See Supplementary Materials Section 1, available as supplementary data at *Bioinformatics* online, for preprocessing and cell type annotation details.

### 3.3 Biological insights

**Figure 2:**
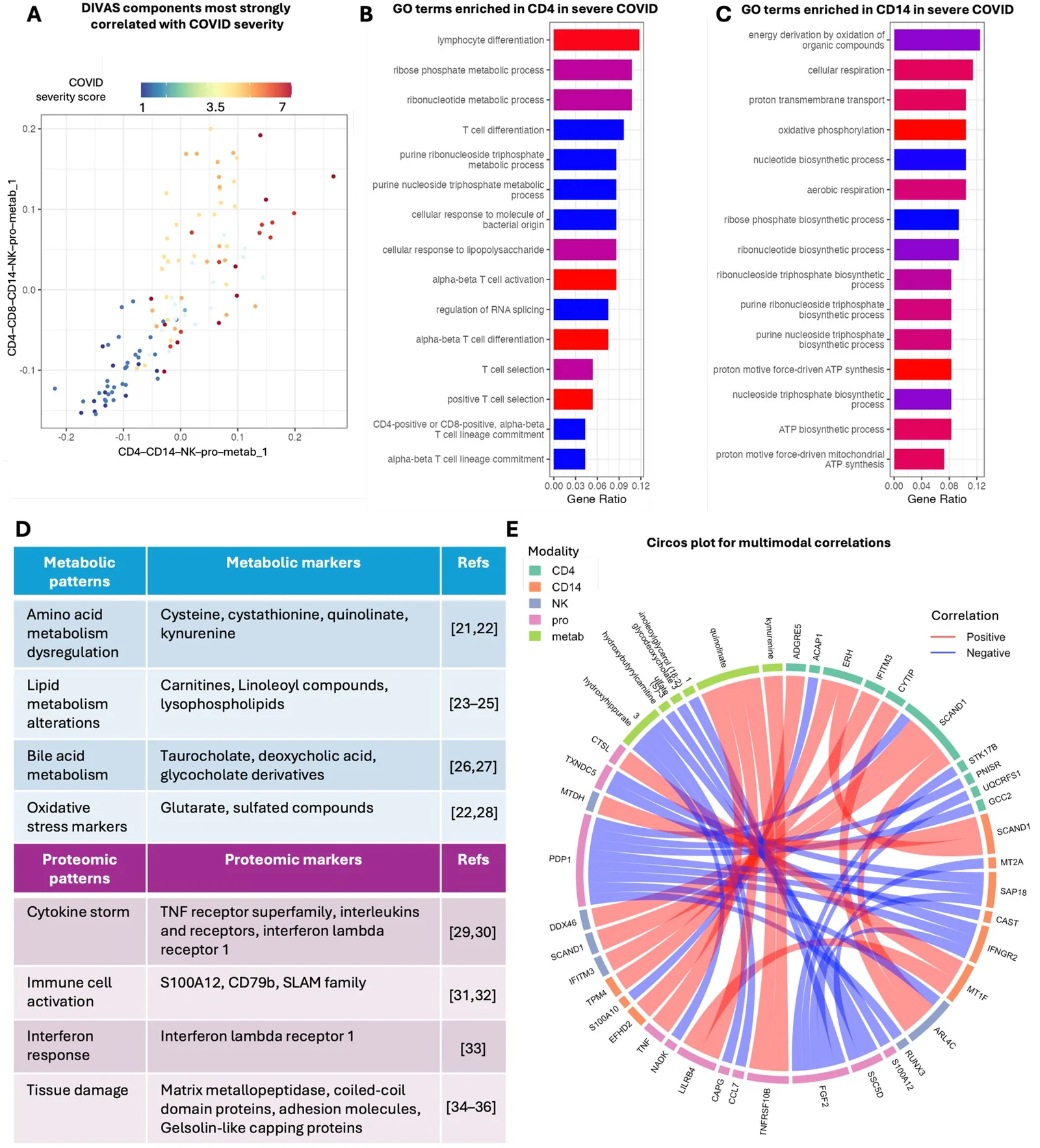
Uncovering multiomic pathways underpinning COVID-19 severity. (a) Visualizing the scores of DIVAS components most strongly correlated with COVID severity. A component, e.g. CD4–CD14–NK–pro–metab_1, is named after the data blocks it represents. As there may be multiple components corresponding to a particular combination of data blocks, we also use an index to distinguish them. (b, c) Gene ontology (GO) terms enriched in CD4 T cell and CD14 monocyte gene expressions in severe COVID-19. (d) Metabolic and proteomic patterns observed in severe COVID-19 (Hojyo *et al*. 2020, Thomas *et al*. 2020, Li *et al*. 2021, Ramasamy and Subbian 2021, Cihan *et al*. 2022, Gelzo *et al*. 2022, Guntur *et al*. 2022, Jackson *et al*. 2022, Labarrere and Kassab 2022, Piñol Jiménez *et al*. 2022, Zanza *et al*. 2022, Iosef *et al*. 2023, Salomão *et al*. 2023, Wei *et al*. 2023, Huang *et al*. 2024, Mester *et al*. 2024). (e) Circos plot illustrating within- and cross-modal correlations, where the thickness of ribbon represents the absolute value of pairwise Spearman correlation coefficient between connected features.

Applying DIVAS to the six-modal COVID-19 data, we found over 90 statistically significant components, ranging from six-way (jointly shared variation) down to one-way (individual variation) (see Fig. 1, available as supplementary data at *Bioinformatics* online). We then ranked all DIVAS component according to the correlation between their scores with the COVID-19 severity score, an ordinal variable ranging from 1 to 7. The top two most highly correlated components are visualized in Fig. 2a (see Fig. 2, available as supplementary data at *Bioinformatics* online, for the top five components).

DIVAS loadings rank modality-specific features by their contribution to a component of interest. For CD4–CD14–NK–pro–metab_1, the component most strongly correlated with severity, we took the CD4 GEX features most positively and negatively associated with severity and conducted gene set overrepresentation analysis (Supplementary Methods Section 2, available as supplementary data at *Bioinformatics* online). The results showed marked differences in CD4 T cell phenotype between severe and mild COVID-19: mild patients showed a more coordinated immune response, marked by T cell and hematopoiesis regulations (Fig. 2b), whereas severe patients showed a highly activated but dysfunctional CD4 T cell state, marked by metabolic stress (Fig. 2c). From the proteomics and metabolomics modalities, we found markers representing a series of immune and metabolic dysregulations (Fig. 2d), confirming the CD4 T cell dysregulation seen in Fig. 2b and c from complementary omics perspectives. The pathways in Fig. 2b–d are well documented in severe COVID-19; recovering them is a positive control that the decomposition is biologically sound. What DIVAS reveals is how these pathways are shared: these programs emerged not as a jointly shared signal across all six blocks, but as a single partially shared component spanning CD4 T, CD14 monocyte, NK, proteomic, and metabolomic blocks while excluding CD8 T cells. A method restricted to jointly shared variation would have to attribute this signal to all six blocks or to none.

Finally, we identified the top features from all five modalities involved in the CD4–CD14–NK–pro–metab_1 component and examined their correlations (see Supplementary Methods Section 2, available as supplementary data at *Bioinformatics* online). Interestingly, we identified correlation structures that helped explain the immune dysregulation mentioned above. Namely multiple chemokines (e.g. CCL7), TNF receptors (e.g. TNFRSF10B), and metabolites (e.g. quinolinate) formed an interconnected network (Fig. 2e), suggesting the cytokine storm that is commonly observed in severe COVID-19 immune response (Hojyo *et al.* 2020, Ramasamy and Subbian 2021, Zanza *et al.* 2022). Another highlight was the multiple strong negative correlations identified across modalities (blue links in Fig. 2e). For example, PDP1 protein was found to be negatively correlated with IFNGR2 gene in CD14 monocytes. This suggested a discordance in severe COVID-19 patients between myeloid activation, for which IFNGR2 is essential (Todorović-Raković and Whitfield 2021), and energy production via oxidative metabolism, promoted by PDP1 (Kumar and Greenberg 2025).

## 4 Discussion

We introduced the DIVAS R package, making available to the bioinformatics community a framework that systematically identifies jointly shared, partially shared and individual variations across multiple data blocks. This fills a critical gap: many biological mechanisms are reflected in only some molecular modalities, and methods restricted to fully shared or individual variation miss these intermediate patterns. Our benchmark confirms this concretely, with neither AJIVE nor MOFA + reliably recovering the partially shared components that DIVAS identified correctly across almost the entire noise range tested.

The properties underpinning this performance are: (i) hierarchical search through all combinations of modalities using angle-based subspace analysis, (ii) built-in inference through principal angle analysis and rotational bootstrap, (iii) simultaneous consideration of sample and feature spaces, and (iv) handling of high-dimensional data through optimal shrinkage.

DIVAS is complementary to probabilistic latent factor models such as MOFA+ (Argelaguet *et al.* 2020) and deep generative frameworks such as MultiVI (Ashuach *et al.* 2023) and MOVE (Allesøe *et al.* 2023). These are preferable when the goal is large-scale nonlinear representation learning, imputation, batch correction, or prediction. DIVAS is preferable when the question is which modalities share a signal, and the answer must be explicit, interpretable, and accompanied by subset-specific ranks, scores, loadings, and inference. The two can be combined: DIVAS loadings offer a structure-aware way to prioritize features from biologically relevant components before predictive modeling, in place of generic filters such as variance thresholding or univariate selection.

Two practical considerations should guide use. First, stable decomposition depends not only on sample size but also on noise, feature filtering, preprocessing quality and the stability of the initial rank estimation, so we do not recommend a universal minimum sample size. Our simulations and case study indicate that DIVAS suits matched studies with roughly 100–300 samples and three–six blocks, with stability checked by repeated runs and perturbation diagnostics near those limits. Notably, DIVAS also yielded interpretable results in preliminary analyses of a four-omics dataset comprising 19 samples, although these analyses are not presented here. Runtime of DIVAS grows with the number of blocks, as the candidate subsets grow combinatorially. Second, normalization and centering affect each block’s relative contribution and hence the estimated subspaces: DIVASmain exposes colCent and rowCent, both FALSE by default, with row-centering appropriate when feature-level mean differences should be removed beforehand. Since DIVAS assumes blocks measured on the same samples in the same order, reproducibility also depends on careful block construction upstream; our vignettes document this workflow end to end, including the diagnostics to inspect before interpreting components.

The package is designed for researchers without specialized expertise in matrix perturbation theory or convex optimization, providing straightforward functions for decomposition and downstream interpretation. As multiomics studies grow more common and more complex, DIVAS offers a principled way to extract the full spectrum of biological information encoded across complementary molecular measurements.

## Supplementary Material

btag615_Supplementary_Data

## Data Availability

The raw scRNA-seq count tables for the COVID-19 case study can be accessed by Array Express under the accession number E-MTAB-9357; metabolomic and proteomic datasets are available from Mendeley Data (https://data.mendeley.com/datasets/tzydswhhb5/5) (Su 2020). Processed data used in this paper are available from Zenodo (Sun 2025).

## Code availability

The DIVAS R package is available on GitHub with function reference documentation and vignettes: https://byronsyun.github.io/DIVAS/. The pipeline used for processing COVID-19 data and the code for reproducing the results in this paper are available on GitHub https://github.com/ByronSyun/DIVAS_COVID19_CaseStudy.

## Use of large language model

For generating the summary of metabolic and proteomic pathways in Fig. 2d, Claude Sonnet 4.5 was used to identify potential pathways based on features with top DIVAS loading values. These pathways were then validated by a thorough literature search and review. We also used Claude Opus 4.8 and ChatGPT 5.6 Sol to check grammatical mistakes and textual inconsistencies.
